# Coincident Fluorescence‐Burst Analysis of Actin Cargo Molecules in Secreted Single Diffusing Extracellular Vesicles From Human Induced Pluripotent Stem Cells

**DOI:** 10.1002/advs.202514421

**Published:** 2025-12-29

**Authors:** Dang Du Nguyen, Aleksandr Barulin, Won Jong Yu, Jong‐Chan Park, Inki Kim

**Affiliations:** ^1^ Department of Biophysics Institute of Quantum Biophysics Sungkyunkwan University Suwon Republic of Korea; ^2^ Department of Intelligent Precision Healthcare Convergence Sungkyunkwan University Suwon Republic of Korea; ^3^ Moscow Center for Advanced Studies Moscow Russia; ^4^ Department of MetaBioHealth Sungkyunkwan University Suwon Republic of Korea; ^5^ Department of Biopharmaceutical Convergence Sungkyunkwan University Suwon Republic of Korea

**Keywords:** actin, Alzheimer's disease, coincident‐burst analysis, extracellular vesicles, fluorescence correlation spectroscopy

## Abstract

Extracellular vesicles (EVs) mediate cellular communication via cargoes of nucleic acids, proteins, and miRNAs, and play key roles in neurodegeneration. However, quantification of specific cargo changes in EVs is challenging due to their small size and heterogeneity. Here, we develop a two‐color fluorescence cross‐correlation spectroscopy (FCCS) and coincident‐burst analysis platform to quantify actin‐enhanced green fluorescent protein and micro red fluorescent protein dual‐labeled EVs secreted from human induced pluripotent stem cells in undifferentiated, partially differentiated, and amyloid beta‐treated conditions. First, a two‐color time trace with pulsed interleaved excitation reveals a markedly increased actin green fluorescent protein burst frequency in EVs derived from partially differentiated cells compared with undifferentiated ones, suggesting altered secretion dynamics under stress. Second, FCCS analysis directly confirms the loading yield elevation based on coincident‐burst analysis of single EVs derived under a partially differentiated condition, indicating enhanced packaging of actin cargo into EVs. Third, the proposed methodology directly quantifies the EV size and the number of actin molecules carried to serve as biogenesis patterns linked to neurodegenerative pathology. Altogether, our platform offers a quantitative, highly specific tool to monitor cytoskeletal disruption via EVs and uncover molecular changes in neuronal differentiation, which is critical for developing therapies for neurological disorders.

## Graphical abstract

This study presents a two‐color FCCS and coincident‐burst analysis platform to quantify actin cargo in dual‐labeled EVs secreted from hiPSCs. The approach tracks secretion dynamics, confirms actin loading, and measures EV size and cargo number, offering insights into cytoskeletal disruption and neuronal differentiation relevant to neurodegenerative disease mechanisms.

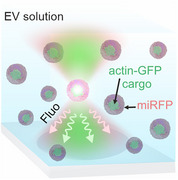

## 1 Introduction

Extracellular vesicles (EVs) are small membrane bound vesicles, typically ranging in size from 30 to 200 nm [1], secreted from living cells that can be readily obtained from urine, plasma, or serum [2]. They are increasingly recognized as biomarkers for revealing the pathogenesis of multiple diseases, including neurodegenerative and cardiovascular diseases and cancers [3, 4, 5, 6, 7, 8] through minimally invasive liquid biopsy. Human induced pluripotent stem cells (hiPSCs) are pluripotent reprogrammed stem cells that have emerged as a powerful tool in regenerative medicine and disease modeling. Owing to their pluripotency, hiPSCs can differentiate into various cell types, making them a more versatile research model than primary cell cultures or immortalized cell lines. Moreover, EVs contain a wide array of biomolecules, including lipids, proteins, and nucleic acids (such as mRNA and miRNA) [8], which play an important role in intercellular communication. Because of their pluripotent nature, hiPSCs are influenced by EVs to maintain their undifferentiated state [9]. In the case of neurons, which actively engage in cell‐to‐cell communication, EVs have been shown to mediate neurotransmitter transfer, contributing to neural signal transmission. Therefore, EVs are important components of intercellular communication [10, 11].

Cytoskeletal proteins, e.g., actin, can also be packaged into EVs [12, 13], suggesting that they may contribute not only to structural organization within the cell but also to signal transduction and the maintenance of cellular properties. Actin, a key regulator of cell shape and dynamics [14]. Moreover, hiPSCs undergo spontaneous differentiation even in the absence of specific external stimuli. This implies that changes in actin expression may reflect the state or differentiation trajectory of hiPSCs [15]. In addition, EVs may influence the progression of Alzheimer's disease (AD) since they can disseminate toxic proteins and trigger neuronal degeneration [16]. Actin dynamics are essential for synapse formation, migration, and axonal outgrowth in neurons [17]. Disruption or excessive stabilization of the actin network has been observed in various neurodegenerative diseases [18]. For instance, in AD, pathological proteins, such as amyloid beta (amyloid‐β) and tau, disrupt the function of actin‐binding proteins, leading to the formation of cofilin‐actin rods, which in turn interfere with synaptic signaling and induce cell death [19, 20]. When pathological EVs containing proteins, such as amyloid‐β or tau, are transferred to the surrounding healthy cells, they may accelerate disease progression. However, the relationship between actin and EVs under different culture conditions or during hiPSC differentiation remains poorly understood. Therefore, monitoring actin dynamics via EVs could provide valuable insights into cellular states and serve as an indicator of disease progression in pathological models.

EV cellular communication makes it possible to explain pathological conditions based on the cargo molecule content, concentration, EV phenotype, and size [13, 21, 22, 23]. Therefore, sensitive or reliable methods for accurately probing the size and content of specific EVs in real‐time could benefit from accurate diagnostic approaches. Optical fluorescence microscopy has emerged as an effective method for capturing EV phenotyping through enriched membrane‐associated protein labeling and cargo molecule labeling [24]. Although visible‐light microscopy is ideal for imaging living cells, its resolution is ultimately limited by diffraction, hindering direct spatial visualization of size [25], and it has insufficient sensitivity for assessing low EV sizes or cargo molecule numbers [26]. In contrast, single‐molecule time‐resolved spectroscopy may reveal dynamic systematic events, such as diffusion and molecular interactions [27, 28]. In the context of single‐EV characterization, single‐molecule fluorescence imaging makes it possible to characterize bleaching steps from molecules tagged to immobilized EVs to count single proteins on the surface [29, 30]. However, the immobilization or trapping of EVs requires additional sample preparation time and complex setup settings and lacks access to size characterization and real‐time monitoring of expressed EVs [31, 32]. In parallel, plasmonic nanoantenna platforms have emerged as powerful tools to study vesicle‐mediated cellular communication with single‐vesicle sensitivity. By coupling molecular absorbers to metallic nanoantennas and reading out plasmon resonance energy transfer in dark‐field scattering, these systems enable long‐term monitoring of enzymatic activity associated with vesicle release and organelle function, as demonstrated for oscillatory enzyme azoreductase activity in outer membrane vesicle‐mediated bacterial communication in individual bacteria and for hypoxia‐dependent azoreductase activity at the single‐organelle level [33, 34]. These approaches highlight the potential of nanophotonic probes for monitoring dynamic molecular cargo and vesicle‐related signaling in situ, but they do not directly quantify the number of secreted EVs and the number of specific protein cargo molecules carried by each vesicle.

Single‐color fluorescence correlation spectroscopy (FCS), a dynamic platform for sensing single molecules, has been employed extensively in EV research to achieve single‐particle quantification of vesicle size, concentration, and molecular interactions [35, 36, 37]. Owing to the precisely controlled collection spot, FCS can deliver versatile and high‐throughput analysis of multiple isolated EV types at a single‐vesicle level [36, 38, 39]. Size determination helps determine the analyzed dominant EV type, the quality of the isolation procedure, and aggregate presence [21, 35, 40]. However, the conventional single‐color FCS of EVs is compromised by high background fluorescence from freely diffusing, unincorporated fluorophores, which mask true vesicle‐associated signals and undermine quantitative precision [41]. Fluorescence cross‐correlation spectroscopy (FCCS) monitors synchronized fluctuations of two fluorescent labels to detect nanoscale diffusion, interaction, and single‐molecule brightness. This approach is widely used for the dynamic molecular monitoring of live cells [42]. Moreover, it was shown recently that exosome‐mimetic vesicles can be quantified using this method to detect the presence of cargo molecules inside single nanovesicles [43]. In particular, Briffault et al. [44]. used FCCS to confirm membrane coating of polymeric nanoparticle cores with EV‐derived membranes, thereby validating the core–shell structure of a biomimetic drug delivery platform. Their study addressed nanoparticle formulation and membrane fusion but did not investigate native EVs or their internal cargo content. Nevertheless, to the best of the authors’ knowledge, two‐color real‐time analysis of EVs secreted at a single cargo molecule has not yet been reported.

In this study, two‐color fluorescence single‐molecule spectroscopy was adapted to specific target molecules for analyzing single EVs secreted by hiPSCs and containing actin‐enhanced green fluorescent protein (actin‐EGFP). The hiPSCs were transfected with a CRISPR‐Cas9 construct to knock in the ACTB‐EGFP gene. To ensure that actin‐EGFP was inherently loaded inside EVs, EVs secreted by actin‐EGFP expressing hiPSCs were isolated using size exclusion chromatography (SEC). The extracted EVs subsequently underwent surface staining with micro red fluorescence protein (miRFP) molecules. Then, perfectly overlapping laser beams at wavelengths of 485 and 635 nm were focused inside the EV solution to extract the fluorescence signal and fast fluctuations arising from EV diffusion through the confocal volume. A pulsed interleaved excitation (PIE) scheme and corresponding fluorescence time gating were employed to minimize crosstalk between the green and red fluorescence detection channels. Because of the single‐molecule sensitivity of FCCS, the number of EVs from undifferentiated and partially differentiated hiPSCs was quantified to investigate thoroughly the number of two‐color‐labeled emitters representing the loaded EVs. Given that nanovesicle samples are inherently heterogeneous and highly diluted, FCCS analysis may overgeneralize single EV events by averaging out the size, EV brightness, and cargo molecule content inside EVs. To capture single‐molecule events directly from the data of photon time tags, in situ individual burst duration and intensity were quantified to determine the distribution of EV sizes and the number of actin‐EGFP molecules inside EVs in real‐time. Burst analysis makes real‐time size distribution measurements possible that agree with the nanoparticle tracking analysis (NTA) method for the entire EV mixture. Owing to the single‐molecule sensitivity and specificity, one can assess the number of loaded EVs and the number of their cargo actin‐EGFP molecules quantitatively. Coincident‐burst analysis makes precise quantification of actin cargo possible under undifferentiated, partially differentiated, and pathological conditions. The increase in coincident‐burst occurrence was monitored under partially differentiated and pathological conditions, and the loading yield allowed direct delineation of EVs expressed from hiPSCs. Quantitative assessment of the content of a single EV in real time can open new perspectives for early ND diagnostics and future therapeutic interventions.

## 2 Results and Discussion

### 2.1 Cell Transfection and Derivation of Actin‐Containing EVs From hiPSCs

**FIGURE 1:**
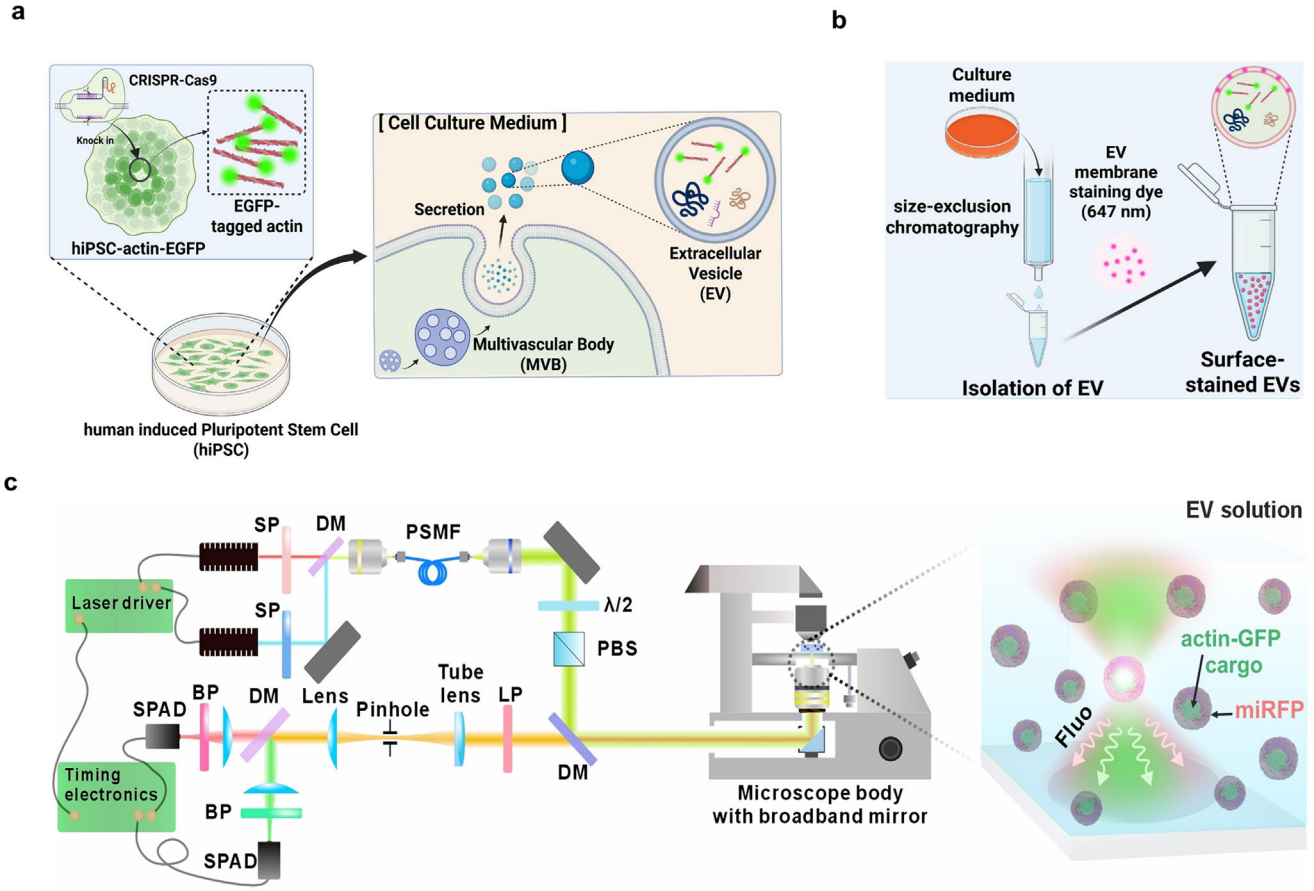
Dynamic monitoring dual‐color single‐molecule platform for quantifying actin in hiPSC‐derived EVs. (a) Generation of human induced pluripotent stem cells (hiPSCs) stably expressing actin‐EGFP. Actin‐EGFP is incorporated into the cytoskeleton of cells and selectively packaged into secreted EVs. (b) Workflow of EV production, isolation, and staining. hiPSC‐actin‐EGFP cells are cultured under undifferentiated, partially differentiated, or amyloid‐β‐treated conditions. EVs are isolated from conditioned media through SEC and stained with miRFP to probe vesicle surface composition. (c) Optical setup for dual‐color fluorescence detection using time‐resolved FCCS. EVs are illuminated within a confocal volume using pulsed lasers, and fluorescence signals from actin‐EGFP (green) and surface‐bound miRFP (red) are separated and recorded by single‐photon avalanche diodes (SPADs) for burst and correlation analysis.

To measure the changes in actin dynamics within EVs, we utilized a human induced pluripotent stem cell line, which carries a CRISPR‐Cas9‐mediated knock‐in of EGFP at the ACTB locus (see Experimental Section). These hiPSCs secreted EGFP‐tagged actin through a process that involves early endosomes, multivesicular bodies, and eventual packaging into EVs (Figure 1a). To examine changes in EVs and actin under different environmental conditions, three experimental groups were established: undifferentiated, partially differentiated, and amyloid‐β‐treated (AD‐associated) conditions. All experiments were initiated when hiPSC cultures reached approximately 50% confluency in six‐well plates and induction of different conditions. In the undifferentiated condition, hiPSCs were cultured in mTeSR Plus medium, which is an optimized medium for maintaining pluripotency. Under this condition, the hiPSCs maintained their optimal morphology and pluripotency. The partially differentiated condition creates an environment in which hiPSCs cannot maintain their pluripotency, thereby inducing spontaneous differentiation. Exposure to suboptimal conditions promotes differentiation, resulting in partial or random lineage commitment. The AD‐associated condition was modeled using amyloid‐β, a pathological hallmark of AD. Among the different forms, oligomerized amyloid‐β is known to be more cytotoxic than the monomeric or fibrillar forms. After culturing under each condition, when the cells reached more than 70% confluence, the culture media were replaced with serum‐reduced Opti‐MEM and incubated overnight to allow for sufficient EV secretion. The culture media were collected, and EVs were isolated using SEC. The EV membranes were labeled with miRFP (647 nm) using a membrane‐staining dye (see Experimental Section), making single EV detection by FCCS possible (Figure 1b). The successful expression of actin‐EGFP by hiPSCs was confirmed through monitoring of bright green emission within the cell volume (Figure S1). Furthermore, the EV size and surface charges were characterized using dynamic light scattering (DLS) and the zeta potential. The EV size distribution was 100–200 nm mostly, and charging was uniform, which agrees with the typical EV characterization [3, 23, 45] (Figure S2). In addition, transmission electron microscopy (TEM) of the isolated actin‐EGFP‐containing EVs revealed well‐defined, round membrane‐bound vesicles within the expected nanometer range and without obvious aggregation, further confirming the successful isolation and morphology of EVs (Figure S3), which is consistent with TEM images of isolated EVs from previous works [46, 47].

Figure 1c shows the custom two‐color, time‐resolved confocal fluorescence microscope setup for the simultaneous detection of internal actin‐EGFP cargo and membrane‐bound miRFP staining in single EVs. After secretion from actin‐EGFP‐transfected hiPSCs and isolation by SEC, EVs were surface‐labeled with cell‐impermeant miRFP. In the microscope body, alternating 485 and 635 nm picosecond laser pulses were guided toward a high‐numerical‐aperture (NA) water immersion objective to focus and excite actin‐EGFP and miRFP molecules sequentially in the same confocal volume. Photon arrival times were recorded using a single‐photon detector connected to a time‐resolved module (see Experimental Section). The photon tag data were analyzed to extract autocorrelation functions in each detection channel, cross‐correlation functions between the channels, and burst parameters (molecule counts) for every individual vesicle.

### 2.2 Dual‐Color FCCS of Single EVs Loaded With Actin‐EGFP

**FIGURE 2:**
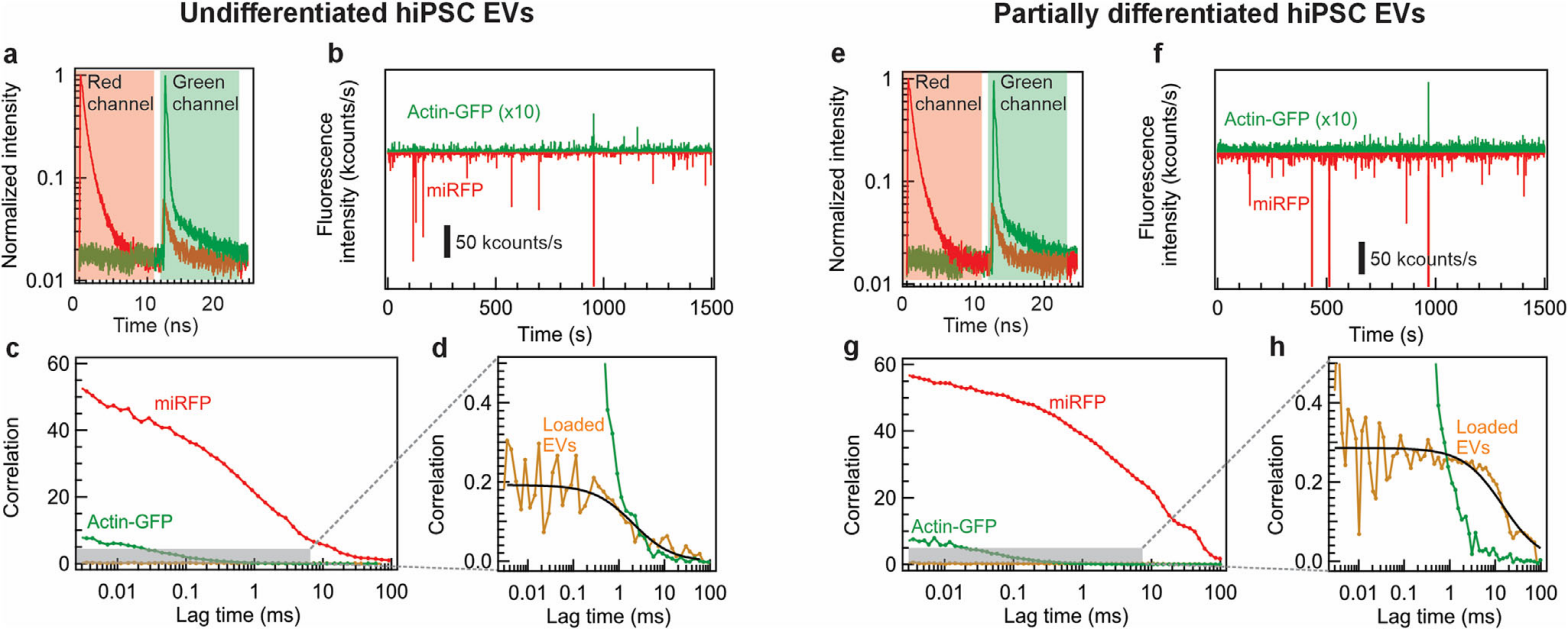
Two‐color fluorescence cross‐correlation spectroscopy of miRFP‐labeled EVs with actin‐EGFP cargo molecules. (a) PIE excitation fluorescence decays of undifferentiated hiPSC EVs derived from red and green detection channels. The shaded red and green regions correspond to the time gates of acquired signals for miRFP and actin‐EGFP emission, respectively. (b) Time‐gated fluorescence time traces in both detection channels of undifferentiated hiPSC EVs. The red fluorescence time trace is inverted, while the green time trace is multiplied by 10 for coincident‐burst representation clarity. (c) Autocorrelation and cross‐correlation functions of the fluorescence signal of undifferentiated hiPSC EVs. (d) Magnification of Panel (c) visualizing cross‐correlation at low amplitude values. (e) PIE excitation fluorescence decays of EVs derived from partially differentiated hiPSCs in red and green detection channels. (f) Time‐gated fluorescence time traces in both detection channels of partially differentiated hiPSC EVs. (g) Autocorrelation and cross‐correlation functions of the fluorescence signal of partially differentiated hiPSC EVs. (h) Magnification of Panel (g) visualizing cross‐correlation at low amplitude values.

To dissect how cellular stress alters the surface and internal composition of single EVs, PIE‐FCCS was applied to EVs isolated from undifferentiated and partially differentiated hiPSCs. Owing to the time‐correlated single‐photon counting (TCSPC) functionality, the photons can be time‐gated according to their excitation pulses. In PIE‐FCCS, alternating 485 and 635 nm picosecond laser pulses by half the synchronization period created temporally separated excitation windows for actin‐EGFP and miRFP, respectively, thereby eliminating spectral crosstalk [48, 49] (Figure 2a). The two lasers generated pulse trains at 40 MHz, interleaved by a 12.5 ns emission start time delay. This delay is negligible relative to the millisecond‐scale diffusion time of an EV through the confocal volume, yet sufficiently longer than the emission lifetimes of the fluorophores. This made the interleaved acquisition of fluorescence intensity traces for each channel and the extraction of their FCCS curves possible. To ensure that the collected photons were retrieved from red and green fluorescent molecules at the same point in the aqueous solution, a multicolor‐stained 110 nm nanoparticle was scanned along all dimensions (Figure S4). The laser focal volumes overlapped perfectly in the lateral plane and overlapped along the optical axis with a shift of 300 nm (less than 15% of the axial beam length *2Z_0_*). The reliability of FCCS was validated by observing unbound molecule mixtures and dual‐color tagged reference nanoparticles. In the case of negligibly low background noise, the bound fraction is given by the ratio of the cross‐correlation and autocorrelation amplitudes at zero lag time (see Text S1). Unbound Alexa Fluor 488 and Alexa Fluor 647 yielded clean autocorrelation functions on both channels, and cross‐correlation was absent. In contrast, multicolor‐stained nanoparticles exhibited a high cross‐correlation amplitude of nearly the same value as those of the autocorrelations (Figure S5), implying a direct pathway for the real‐time characterization of dual‐color single emitters.

Dual‐color FCCS data, including autocorrelation and cross‐correlation functions, were represented by EVs derived from undifferentiated and partially differentiated hiPSCs. Partially differentiated hiPSCs were grown in an unmatched medium to induce stress and overexpression of actin. The rationale for including these hiPSC conditions stems from emerging evidence that partially differentiated cells exhibit stress‐like phenotypes, which may alter cytoskeletal organization and promote differential EV biogenesis [50]. In this context, actin cargo is a critical cytoskeletal protein involved in vesicle trafficking and EV formation, and its dysregulation is increasingly linked to neurodegenerative processes [51]. Previous studies have reported that actin expression is dynamically regulated during iPSC differentiation, and that iPSC‐derived mesenchymal stem cells exhibit enhanced EV secretion compared to adult MSCs [52, 53]. By comparing actin EV secretion between undifferentiated and partially differentiated hiPSCs, we aimed to uncover how stress‐associated differentiation affects cytoskeletal remodeling and EV‐mediated actin export. These underscore the importance of cellular differentiation in EV studies, especially when interpreting EV‐based biomarkers in disease modeling. TCSPC histograms displayed distinct decay profiles in the red (miRFP) and green (actin‐EGFP) detection channels, confirming clean channel separation. Concurrently, EVs derived from undifferentiated hiPSCs yielded pronounced fluorescence bursts that were recorded over time, revealing condition‐dependent changes in both membrane staining and cargo loading (Figure 2b). Here, G_g_ and G_r_ are the autocorrelation functions of actin‐EGFP fluorescence inside the vesicle lumen and miRFP fluorescence on the vesicle membrane, respectively. In contrast, G_rg_ is the cross‐correlation function between these two signals. The raw data of photon time tags are used to reconstruct fluorescence autocorrelation and cross‐correlation functions versus lag time τ from two channels (Figures 2c,d). Because EGFP is endogenously fused with actin, the cytoskeletal components that the hiPSCs naturally sort into EVs were measured precisely, providing mechanistic insights into how actin dynamics influence vesicle biogenesis. The combination of luminal EGFP and orthogonal miRFP surface staining virtually eliminated the background from free dye or non‐vesicular lipid particles, yielding a cleaner search of truly loaded EVs. Although FCCS excels at characterizing particle populations of uniform size and brightness, it can be challenging to interpret when applied to highly heterogeneous and polydisperse vesicle ensembles, as is common for exosomes [54]. Nonetheless, the resulting FCCS data yielded important insights into distinct vesicle subpopulations and their molecular cargoes. The fitting parameters for the FCCS data are listed in Table S1. Nonzero G_rg_(0) values confirmed that actin‐EGFP‐loaded EVs were present in the studied samples. The faster diffusion component of G_g_ matched the behavior of free actin‐EGFP species in solution, demonstrating that residual, unincorporated actin‐EGFP contributed significantly to the green‐channel signal and limited the accuracy of single‐color FCS measurements. The red autocorrelation functions exhibited large amplitudes, indicating that most of the miRFP molecules were bound to the membrane. Fitting fluorescence autocorrelation and cross‐correlation functions with the 3D diffusion model yields:

(1) $$G_{D}\;\left(\tau \right)=\frac{{\left(1-B/F\right)}^{2}}{N}\;\cdot \frac{1}{\left(1+\frac{\tau }{\tau_{D}}\right)\sqrt{1+\frac{\tau \omega_{xy}^{2}}{\tau_{D}\omega_{z}^{2}}}}$$

Here, *τ_D_* is the molecule diffusion time, *B* denotes background noise, *F* is the average fluorescence signal, ω_xy_ and ω_z_ are lateral and axial focal waists, and *N* corresponds to the number of molecules. Regardless of the low background noise in the confocal microscope setup (typically approximately 200 count/s), single‐molecule events were relatively rare, highlighting the low average fluorescence signal, which is comparable to the average background noise. To extract the number of loaded EVs, the number of dual‐color emitters (N_rg_) was quantified using background accounting (see Text S1). The average number of loaded EVs was 1.8 × 10^−4^ in the confocal volume at the instance of time, while the total number of detected actin‐EGFP emitters was 2.3 × 10^−2^. The ratio *N_rg_/N_r_* constitutes the loading yield of actin‐EGFP EVs, which, in the case of undifferentiated hiPSC EVs amounts to 3.1%. In contrast, EVs derived from partially differentiated hiPSCs grown under unmatched medium stress were analyzed to observe the cargo protein changes in EVs. Similarly, time‐gated PIE excitation time traces (Figure 2e) were obtained from the EV signals in both detection channels. The fluorescence time trace from the actin‐EGFP signal was higher than in the case of undifferentiated hiPSC EVs, which became slightly visible in the bare time trace data (Figure 2f). Computing the number of loaded EVs from the autocorrelation and cross‐correlation functions (Figures 2g,h) revealed that partially differentiated hiPSC EVs have a higher actin‐EGFP content with *N_rg_* of 2.8 × 10^−4^ and *N_g_* of 4.2 × 10^−2^ compared with their undifferentiated counterparts. The increased actin‐EGFP expression in undifferentiated hiPSC EVs tended to increase the EV loading yield slightly to 4.4% (see Figure 4 for statistical analysis).

These data demonstrate that unmatched‐medium‐induced stress increases the number of actin‐loaded EVs. This confirms that cellular stress conditions drive coordinated changes in EV surface properties and cytoskeletal cargo loading. This dual‐color FCCS approach offers several important advantages. It resolves heterogeneity by quantifying luminal actin cargo and membrane markers on a vesicle‐by‐vesicle basis, achieves single‐molecule sensitivity, detects as few as one GFP molecule per EV, and simultaneously provides biophysical parameters (molecular occupancy and vesicle size) from microliter‐scale samples without the need for surface immobilization. By combining single‐molecule sensitivity with vesicle‐by‐vesicle sizing and dual‐color readouts, this platform can quantify changes in EV actin content and membrane markers systematically across large sample sets.

### 2.3 Quantification of EV Size and Actin Load Through Coincident‐Burst Analysis

Because each EGFP tag corresponds to one actin molecule, burst amplitudes translate directly into molecule counts per EV. The fluorescence burst analysis makes it possible to retrieve the transit time of each passing EV and the intensity of the burst, yielding not only the average size and brightness values but also the distribution of the corresponding experimental parameters [43]. A burst search library was used to extract green and red fluorescence bursts resulting from actin‐EGFP and miRFP molecule fluorescence, respectively (see Experimental Section). In the case of undifferentiated hiPSC EVs, the burst parameters, including burst threshold value *K·B* and number of consecutive photons contributing in the burst *m*, we adjusted to make the burst duration distribution conform to the FCCS‐determined *τ_D_* results (see Experimental Section). The burst search parameters for the hiPSCs treated under different conditions (Table S2). Given the focal beam waists at both wavelengths, the size of every passing EV was quantified using the Stokes–Einstein diffusion law as follows:

(2) $$EV\;size=\frac{4k_{B}T}{3\pi \eta \omega_{0}^{2}}\;\tau_{burst}$$

**FIGURE 3:**
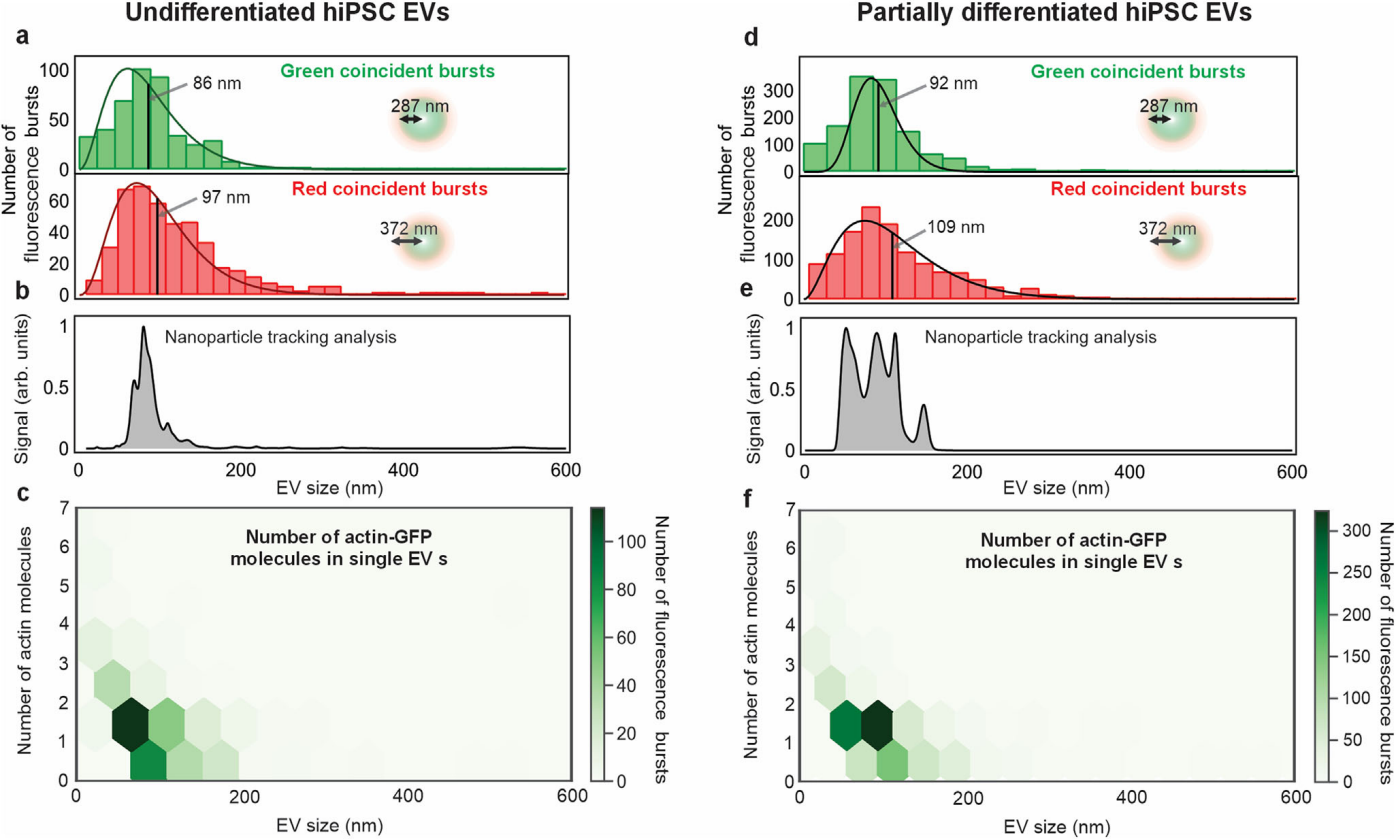
Coincident‐fluorescence‐burst analysis of loaded single actin‐EGFP‐containing EVs. (a) Size distribution of loaded undifferentiated hiPSC EV based on green (top panel) and red (bottom panel) coincident bursts. The inset beam radius values correspond to focal spot waists of green and red lasers. The green and red curves correspond to gamma fits of the size distribution retrieved from burst analysis of green and red coincident bursts, respectively. (b) NTA‐analyzed size distributions of all secreted EVs derived from undifferentiated hiPSC EVs. (c) 2D histograms of EV size and number of actin‐EGFP molecules inside a single undifferentiated hiPSC EV. (d) Size distribution of loaded partially differentiated‐hiPSC‐derived EV based on green (top panel) and red (bottom panel) coincident bursts. The black lines and numbers referred to them in panels (a) and (d) denote the mean values of gamma distribution fit results. The numbers of 287 and 372 nm in panels (a,d) denote the waist of the confocal beam of the green and red excitation light, respectively. (e) NTA‐analyzed size distribution of all secreted EVs derived from partially differentiated hiPSC EVs. (f) 2D histograms of EV size and number of actin‐EGFP molecules inside single partially differentiated hiPSC EV.

Here, *k_B_* denotes the Boltzmann constant, *T* and η are the temperature and viscosity of the solution medium, respectively, ω_0_ denotes confocal beam waists for 485 or 635 nm lasers, and τ_*burst*_ corresponds to the duration of the detected burst in the green or red detection channel. The occurrence times of bursts found in both channels were then cross‐checked to reveal the coincident events based on the burst center time, which matches within a defined tolerance window (see Experimental Section). The identification of coincident bursts made it possible to select a subpopulation of single EVs loaded with actin‐EGFP only. Figure 3a shows the undifferentiated hiPSC EV size distribution. The obtained EV [55] size histograms are fitted with the rescaled gamma distribution function: $f\;\left(x\right)=C\frac{\lambda^{\alpha }}{\Gamma (\alpha )}x^{\alpha -1}e^{-\lambda x},$ where α/λ corresponds to the gamma distribution function mean, Γ(α) is the gamma function. The measured size distributions in both channels agreed well with each other and the results of NTA (Figure 3b). The latter analysis made it possible to validate the applicability of the burst search algorithm. Based on the burst intensity, the number of EGFP‐actin molecules in each loaded EV was quantified approximately. The green coincident‐burst intensity distribution was divided by the independently determined free actin‐EGFP molecule brightness, which approximately represented the number of single actin‐EGFP molecules inside an EV [43]. Figure 3c shows a 2D histogram of a single EV size and actin‐EGFP content retrieved from the burst data. Each hexagonal bin represents the number of detected EV events, with most bursts arising from EVs smaller than 200 nm that contain 1–2 actin‐EGFP molecules. The undifferentiated hiPSC EVs carry 1–2 cargo actin‐EGFP molecules with sizes of about 90 nm. This indicated selective and sparse protein loading in small EV subpopulations. Furthermore, burst analysis was applied to the data of photon tags from partially differentiated hiPSC‐loaded EVs, and the agreement between the coincident‐burst data and NTA data for the EV size distribution was confirmed (Figure 3d,e). The size distribution appears broader but still less than 200 nm. The number of actin‐EGFPs seems not to change significantly under the stressed hiPSC growth condition; nevertheless, the number of coincident bursts increased by two to three times compared with the undifferentiated hiPSCs (Figure 3f).

**FIGURE 4:**
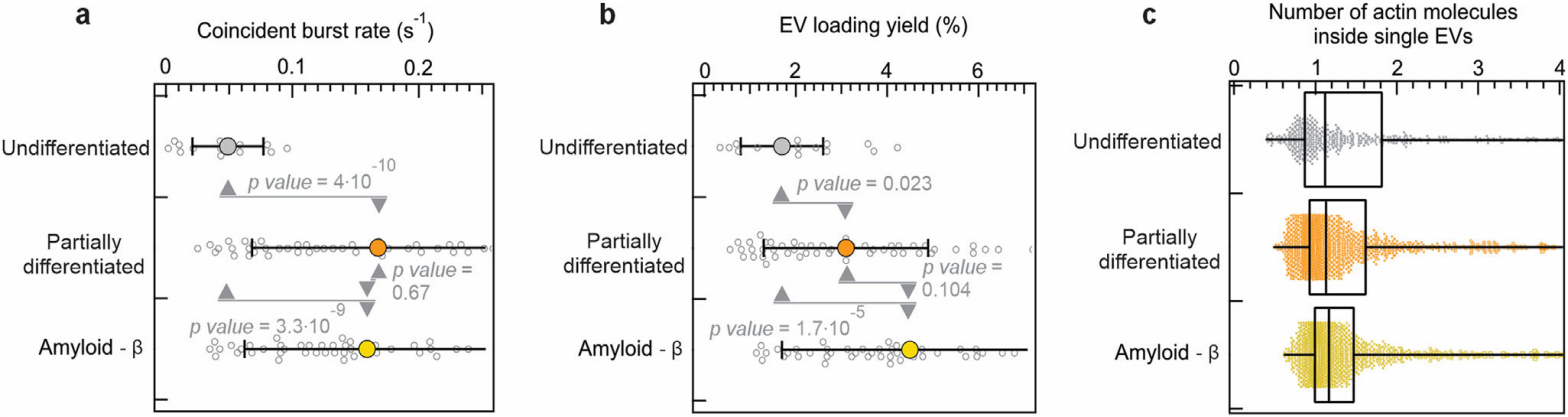
Quantification of actin‐EGFP content in loaded EVs derived from hiPSCs under various treatment conditions based on coincident‐burst analysis. (a) Coincident‐burst rate from single diffusing EVs derived from undifferentiated, partially differentiated, and amyloid‐β‐treated hiPSCs. The *p*‐values correspond to the *t*‐test of the difference between undifferentiated‐hiPSC‐related data and the other two conditions. (b) EV loading yield of EVs derived from undifferentiated, partially differentiated, and amyloid‐β‐treated hiPSCs. The *p*‐values shown in Panels (a) and (b) correspond to the *t*‐test of the difference between undifferentiated‐hiPSC‐related data and the other two pathological conditions. The error bars on panels (a) and (b) correspond to the standard deviation from multiple time trace subsets containing 20 coincident bursts each. (c) Box plot of the number of actin‐EGFP molecules inside single detected loaded EVs that are derived from undifferentiated, partially differentiated, and amyloid‐β‐treated hiPSCs.

Revealing the number of coincident bursts within a time trace made it possible to determine the coincident‐burst rate as a metric of the concentration of loaded EVs. Because each coincident burst marks a single dual‐labeled EV passing through a fixed confocal volume, under constant diffusion conditions, the burst rate is directly proportional to the concentration of loaded EVs. The coincident‐burst rate from undifferentiated hiPSC EVs was 2.7 loaded EVs per minute, whereas this value increased approximately three times in the case of partially differentiated hiPSC EV emission (Figure 4a). Moreover, it was validated that burst analysis revealed that the actin‐EGFP EV expression increased at other hiPSC stress conditions. The undifferentiated hiPSCs were treated with amyloid‐β agent during their growth to induce cell malfunctioning, similarly to the cultured hiPSCs in an unmatched medium. Amyloid‐β is used here to simulate the cellular environment of AD, enabling us to study how EV production or release changes under AD‐like stress. The corresponding FCCS, NTA, and coincident‐burst analysis data are shown in Figure S6. The rate of counted coincident bursts from EVs derived from amyloid‐β‐treated hiPSCs is nearly identical to that of the partially differentiated hiPSC counterparts. Moreover, the statistical *t‐*tests confirm the significance of the difference between the coincident‐burst rates of undifferentiated and partially differentiated/amyloid‐β conditions of hiPSC culturing, as *p*‐values fall below 0.05. By dividing the coincident‐burst rate and total red‐burst rate, it was possible to determine the EV loading yield (Figure 4b). Similar to the FCCS results, the loading yield increased under pathological conditions compared with undifferentiated hiPSC EVs. Finally, EVs from undifferentiated hiPSCs contain one to two actin molecules with low variability, while partially differentiated and amyloid‐β‐treated hiPSCs release more heterogeneous, actin‐enriched EVs (Figure 4c). These findings highlight that cellular stress increases both the number and the variability of actin cargos per EV, where excess or misregulated actin is actively cleared through EVs, possibly as part of a cellular stress response to neurodegenerative insults [51, 56, 57].

## 3 Conclusion

A two‐color FCCS platform combined with coincident‐burst analysis was developed and used to dissect quantitatively the molecular composition and size distribution of actin‐EGFP and miRFP dual‐labeled EVs derived from hiPSCs under different conditions. The approach addresses important challenges in EV research, such as probing size, heterogeneity, determining the quantitative loading information, circumventing the high background fluorescence of nonloaded biomarkers, and performing real‐time analyses. The findings reveal that cellular stress induced by partially differentiated and amyloid‐β exposure substantially alters EV secretion dynamics and cytoskeletal cargo loading. Based on the changes in TSG101 expression observed in both hiPSCs and hiPSC‐derived cerebral organoids (COs), we speculate that EV secretion may have increased indirectly through a TSG101‐independent pathway [58] (see Figures S7 and S8, Supporting Information). It is also possible that TSG101 was preferentially packaged into secreted EVs, leading to a reduction in its intracellular level. These findings suggest that our experimental model may induce fluctuations in EV secretion in response to stress, indicating its potential applicability for studying stress‐related EV dynamics. Although our observations were based on indirect evidence from Western blot analysis, we believe that additional proteomic analysis could provide a more detailed and direct comparison of the similarities and differences in secreted EVs across the experimental conditions. Furthermore, when EVs derived from the partially differentiated and amyloid‐β treated hiPSC groups were applied to a separate healthy hiPSC line, we observed a tendency toward increased differentiation around the colony area (see Figure S9). Although this observation is indirect, it may indicate that EVs have the potential to influence neighboring cells through cell‐to‐cell communication. Specifically, EVs secreted under these conditions exhibited an increased actin‐EGFP burst frequency, higher dual‐color coincidence rates, and enriched actin cargo content, while also displaying shifts toward smaller vesicle sizes. These results align with the emerging evidence that EV biogenesis is tightly linked to cytoskeletal remodeling and cellular stress responses [56, 59, 60, 61]. Amyloid‐β treatment relevant to AD enhanced the secretion of actin‐enriched EVs, providing potential insights into the mechanisms by which cytoskeletal disruptions propagate in neurodegenerative disorders [51, 57].

Although the dual‐color FCCS platform combined with coincident‐burst analysis provides single‐vesicle precision for quantifying EV cargo loading and size, several limitations restrict its broader applicability. The technique requires fluorescent labeling of both EV membranes and internal cargo molecules, thereby limiting clinical biomarker studies. The methods are performed within a small confocal detection volume, allowing single‐vesicle resolution but are limited by the concentration requirements. As extremely low EV concentration (femtomolar or attomolar range) would lead to excessively long waiting times between single‐EV detection events for a satisfactory statistical analysis. Possible potential strategies to overcome this limitation is to enlarge the detection volume [62] or to increase the effective throughput by integrating high‐throughput FCS and automated screening [36]. Their approach enables spatial localization and transient trapping of EVs that can be continuously monitored. This not only reduces the required acquisition time but also increases the overall detection efficiency.

Our study has several important limitations that should be acknowledged. All experiments in this study were conducted in vitro using hiPSC‐derived EVs, without testing their behavior in vivo models. Consequently, cargo stability and uptake mechanisms within living tissues remain to be established. Furthermore, the analysis focused solely on actin‐EGFP as a cytoskeletal model cargo. Other relevant biomolecules, such as RNA, tau, or α‐synuclein, were not examined, which limits the generalizability of the results to broader EV‐mediated processes. However, recent development demonstrates the powerful capabilities of FCS for detecting low‐abundance pathological aggregates like α‐synuclein at single‐molecule resolution [63]. This method offers critical insight into protein aggregation dynamics and diffusion behavior in complex biological solutions. When applied to EV research, such FCS‐based techniques including dual‐color FCCS along with coincident burst analysis provide a means to track the packaging, association, and release of pathological proteins (e.g., α‐synuclein or tau) via EVs. Coincident burst analysis enables the detection and quantification of single EVs carrying specific pathological molecules, while changes in diffusion properties or cross‐correlation amplitudes from FCCS can indicate aggregation states or cargo unpacking. This approach holds great promise for elucidating mechanisms of EV‐mediated intercellular propagation of neurodegenerative disease‐associated proteins and for monitoring their trafficking, loading efficiency, or degradation dynamics under disease‐mimicking conditions.

One promising application of FCCS is optimizing EV‐based drug delivery systems [64], where precise characterization of vesicle size, surface properties, cargo loading, and stability is essential. Efficient encapsulation is a critical first step to ensure that therapeutic molecules remain protected throughout transport and are successfully delivered to target cells. When cargo molecules are fluorescently labeled, their release can be tracked by detecting faster‐diffusing species in the confocal volume. However, once released, these molecules may bind to cellular components, altering their diffusion behavior. To obtain more accurate release data, labeling both the cargo and nanocarriers with distinct fluorophores and monitoring their colocalization using FCCS. This approach has been effectively demonstrated in previous studies [65], such as the use of FCCS to characterize the assembly and release behavior of cationic liposome‐DNA nanoparticles, where co‐diffusion analysis provided quantitative insights into binding efficiency and complex stability without requiring separation steps. Such dual‐color FCCS strategies could be particularly valuable in tracking the intracellular fate of therapeutic messenger RNA (mRNA) formulations, as highlighted in a recent study [66] demonstrating that polycations can enhance the cytosolic stability of mRNA delivered by lipid nanoparticles. By simultaneously labeling both the mRNA cargo and nanocarrier components, FCCS allows direct observation of release events, intracellular unpacking, offering a complementary biophysical readout to assess how polycationic additives influence mRNA delivery kinetics and duration of protein expression. While these previous studies focused on nanocarriers like liposomes or lipid nanoparticles, the same FCCS principles can be extended to EVs, which share key structural and biophysical properties. Since EVs are naturally occurring lipid bilayer vesicles, fluorescent labeling of therapeutic cargo (e.g., RNAs or proteins) and EV membranes would allow FCCS to quantify cargo loading, EV size, and release kinetics at the single‐vesicle level.

Recent work using FCS to study cell‐derived vesicles has shown that biologically sourced lipid vesicles can be efficiently loaded with therapeutic and genetic cargos and tracked both in vitro and in vivo [67]. These vesicles, similar to EVs in structure and targeting ability, demonstrated successful cargo delivery to tumor xenografts in a mouse model. FCS was used in this work to determine the concentration and yield of cell‐derived vesicles generated via nitrogen cavitation from A549 cells. The vesicles were fluorescently labeled with DiI, a dye that lights up when embedded in lipid membranes. By analyzing fluorescence intensity fluctuations caused by vesicles diffusing through the laser focus, the authors extracted key parameters such as vesicle diffusion time and concentration from the FCS autocorrelation curve. This enabled them to estimate a vesicle yield of ∼1.3 × 10^11^ vesicles from 40 million cells, demonstrating high production efficiency. While that study focused on FCS measurements, FCCS extends this capability by enabling dual‐color detection of membrane and cargo within single vesicles, offering a more precise tool to assess therapeutic loading and release dynamics in EV‐based delivery systems.

In summary, the proposed dual‐color FCCS and coincident‐burst analysis platform is a powerful tool for dissecting the molecular complexity of EVs at the single‐vesicle level. The results of this study not only provide mechanistic insights into EV biogenesis and cytoskeletal cargo dynamics but also lay the foundation for future research aimed at leveraging EVs as biomarkers for therapeutic targets in ND [8, 68, 69].

## 4 Experimental Section

### 4.1 Cell Culture to Secrete EVs Under Different Conditions

A monoallelic mEGFP‐tagged ACTB WTC hiPSC line (AICS‐0016, Coriell Institute, USA) was used in this study. hiPSCs were cultured in an mTeSR Plus medium (#100‐0276, STEMCELL Technologies, Canada) to maintain pluripotency. When cultures reached ∼70% confluency, hiPSCs were subcultured as follows. First, mTeSR Plus medium was aspirated from a 100 mm cell culture dish, which was then washed with 8 mL sterile DPBS and aspirated again. Next, 4 mL ReLeSR (enzyme‐free passaging reagent; #100‐0483, STEMCELL Technologies, Canada) was added, and the dish was left at room‐temperature for 30 s. After aspirating most of the ReLeSR (leaving a thin‐film), the dish was incubated at room‐temperature for 5 min. At 5 min 30 s, the dish was transferred to a 37°C, 5% CO_2_ incubator for an additional 2 min. At 7 min 30 s, the dish was removed, and all remaining ReLeSR was aspirated. Finally, 4 mL mTeSR Plus medium was added to detach the cells, which were collected into a 15 mL conical tube, centrifuged at 300 × g for 3 min, and then gently resuspended in fresh mTeSR Plus. For environmental modulation, hiPSCs were seeded in 12‐well plates precoated with Corning Matrigel Growth Factor Reduced Basement Membrane Matrix, free from lactate dehydrogenase elevating virus (Corning, #354230). hiPSCs were seeded at 2 × 10⁵ per well in 1 mL of mTeSR Plus medium. Upon reaching more than 50% confluency, media were switched according to conditions, such as undifferentiated continued in mTeSR Plus and partially differentiated cultured in Dulbecco's Modified Eagle's Medium only.

For the AD‐associated condition, amyloid‐β (4014447, Bachem, Switzerland) was initially solubilized in hexafluoroisopropanol to create monomers and then incubated at room‐temperature for 72 h with shaking (50 rpm). The solution was aliquoted into 50 µL portions and lyophilized using a Savant SpeedVac Integrated Vacuum Concentrator (SPD1030, ThermoFisher, USA). For oligomerization, lyophilized amyloid‐β was dissolved in anhydrous dimethyl sulfoxide to 1 mm and diluted with Dulbecco's Phosphate Buffered Saline to 0.1 mm. This was incubated at 4°C overnight (∼18 h). When hiPSC confluency reached 50%, the 0.1 mm amyloid‐β solution was diluted in mTeSR Plus to a final concentration of 2 µm and applied to the cells. All conditions were cultured overnight in Opti‐MEM to prepare for exosome collection. For the EVs treatment and validation of EVs characteristics, every single batch was composed with at least a triplicate of each condition.

### 4.2 EV Isolation and Membrane Staining

Exosomes were isolated from the culture medium using qEV Single 35 nm Gen2 Columns (ICS‐35, IZON, New Zealand) using the size exclusion chromatography technique. The culture medium was centrifuged at 3000 g for 10 min at room‐temperature, and the supernatant was then collected to remove debris. Exosomes were isolated according to the manufacturer's protocol. The isolated exosomes were concentrated using Nanosep 30k centrifugal filters (OD030C33, Pall Corporation, USA) by centrifugation at 3000 g for 10 min at room‐temperature and stained with miRFP using ExoSparkler Exosome Membrane Staining Dye (EX03, Dojindo, Japan) according to the manufacturer's instructions. A total of 100 µL of labeled exosomes were collected.

### 4.3 Transmission Electron Microscope of Isolated EVs

Following the isolation of actin‐EGFP EVs, the vesicles were resuspended in 100 mm sodium cacodylate buffer. Approximately 3 µL of this suspension was applied onto a carbon‐coated, 300‐mesh copper TEM grid (Ted Pella, Inc., Redding, CA, USA). Once the drop had partially air‐dried, the grid was transferred onto a droplet of fixative (2.5% glutaraldehyde in 100 mm sodium cacodylate buffer, pH 7.0) for 1 min at room‐temperature to preserve the structure of EVs during preparation and imaging observation. The grid was then rinsed by gently touching it to the surfaces of three separate drops of distilled water, and residual liquid was wicked away using filter paper.

The EVs were subsequently stained with 2% aqueous uranyl acetate (98%, ACS Reagent, Polysciences, USA) for 1 min to enhance the contrast of EVs. Excess stain was removed by touching the grid's edge to filter paper, and the grid was allowed to dry completely. Imaging was carried out using a JEOL ARM 200F transmission electron microscope (JEOL, Tokyo, Japan).

### 4.4 hiPSC‐derived Cerebral Organoid Generation

Human cerebral organoids (COs) were generated from hiPSCs following a protocol similar to that used for iPSC subculture. Single iPSCs (1.5 × 10⁶ cells) were seeded into AggreWell 800 plates (34811, StemCell Technologies, Canada) and centrifuged to induce embryoid body (EB) formation (Day 0). Cells were cultured in EB medium supplemented with Y‐27632 (ab144494, Abcam, UK) at 37°C and 5% CO_2_, which was replaced with EB medium without Y‐27632 on day 1.

From days 2 to 4, cells were cultured in induction medium containing dorsomorphin (10 µm), SB431542 (10 µm), and 2‐mercaptoethanol (0.1 mm) in DMEM/F‐12 with GlutaMAX, 20% KnockOut Serum Replacement, 1% MEM Non‐Essential Amino Acids, and penicillin/streptomycin. On day 6, the medium was switched to Neurobasal‐A supplemented with GlutaMAX, B‐27 (without vitamin A), Pen/Strep, and additionally enriched with 0.5% Matrigel, EGF, and FGF. Aggregates were transferred to 100 mm Petri dishes containing 14 mL of NB+ medium and cultured on an orbital shaker (90 rpm) overnight.

On day 7, EBs were transferred to 96‐well ultra‐low attachment U‐bottom plates (7007, Corning) and maintained in NB+ medium with Matrigel, EGF, and FGF. Media were changed daily until day 15, and every other day until day 24. From days 25 to 42, NB+ medium was supplemented with BDNF (20 ng/mL) and NT‐3 (20 ng/mL) and changed every other day. From day 43 onward, each CO was placed in 24‐well ultra‐low attachment plates (3473, Corning) with 500 µL of NB+ medium, refreshed every four days. COs older than day 80 were considered mature and used for experiments.

### 4.5 Western Blotting

During the procedure, COs were maintained on ice to preserve protein integrity. After amyloid‐β‐treatement, samples were washed three times with DPBS and subsequently lysed in RIPA buffer supplemented with a protease and phosphatase inhibitor cocktail (PPPi; P8340, Merck, Germany). The lysates were homogenized, sonicated, and centrifuged at 13 000 rpm for 4°C to collect the supernatant fraction. Protein levels were quantified using a BCA assay. For electrophoresis, 6 µg of protein (diluted to a final volume of 18 µL with RIPA+PPPi, sample buffer, and protein stock) was denatured at 95°C for 5 min and loaded onto Bolt Bis‐Tris gels (NW001505, Thermo Fisher Scientific, USA) together with Novex Sharp Pre‐stained Protein Standard (LC5800, Thermo Fisher Scientific, USA). Gels were run at 200 V for 22 min and proteins were transferred to PVDF membranes using a semidry system (100 V for 1 h). Membranes were blocked with 5% skim milk in TBST, incubated with primary antibodies overnight at 4°C, washed, and then exposed to HRP‐conjugated secondary antibodies. Signal detection was performed using ECL substrate, and images were acquired with an iBright imaging system (Thermo Fisher Scientific, USA). In canse of EVs, the EVs were concentrated with a previously mentioned, then added same volume of RIPA buffer to purified EVs solution.

### 4.6 Nanoparticle Tracking Analysis

The prepared exosomes were characterized using the Malvern Nanosight NS300 system. Fluorescence from actin and general exosome markers was used to analyze the concentration and size distribution. Laser modules with wavelengths of 488 and 642 nm were employed. The exosome samples were diluted 20‐fold in phosphate‐buffered saline (final volume: 1 mL) for analysis. Between samples, laser modules and chambers were cleaned with 70% ethanol. After measurement, the samples were immediately recovered and used for DLS analysis.

### 4.7 Dynamic Light Scattering Analysis

The recovered exosomes were further analyzed using the Malvern Zetasizer Lab. The size distribution and zeta potential were measured using 12 mm square glass cells (PCS1115, Malvern) and Folded Capillary Zeta Cells (DTS1070, Malvern), respectively.

### 4.8 Dual‐Color FCCS Optical Setup

Dual‐color FCS measurements were performed using a custom‐built confocal microscope configured for time‐resolved single‐molecule detection. The optical system was integrated into an inverted microscope (Nikon Eclipse Ti2) and equipped with a high‐NA water immersion objective (Nikon PLAN APO VC, NA 1.2, 60x) and 50 µm pinhole. Excitation was achieved using two picosecond‐pulsed diode lasers (PicoQuant, LDH‐D‐C‐485 for green channel and PicoQuant, LDH‐P‐C‐635 m for red channel), each operating at 40 MHz and a power of 50 µW in PIE mode to minimize spectral crosstalk during cross correlation. The laser driver (PicoQuant Sepia PDL 828) governed both the laser repetition rate of the laser and its synchronization. Each laser beam first passed through a short‐pass filter (Thorlabs, SP500 for the blue laser and SP650 for the red laser) to remove any long‐wavelength fluorescence. The beams were then combined using a dichroic mirror (DM1) (Chroma, ZT561rdc) that overlapped the blue (485 nm) and red (635 nm) lasers. The light signals from each channel were then coupled to polarization‐maintaining single‐mode fibers (PSMFs), which made perfect spatial overlap of the two beams possible. The combined beam was directed by a DM2 (Chroma, ZT488/640rpc) so that it could reflect red and blue lasers and transmit fluorescence light excited into the back aperture of a high‐NA water immersion objective. This produced a diffraction‐limited excitation volume during sample observation. The fluorescence emission was collected through the same objective lens and directed through a long‐pass filter (Semrock, BLP01‐635R‐25), tube lens (Thorlabs, TTL200‐A), and confocal pinhole. After passing through the pinhole, the fluorescent light was collimated by a lens and split into green and red channels by the DM3 (FF649‐Di01‐25 × 36). The emission light from each channel passed through a bandpass filter (Semrock, FF01‐679/41‐25 for the red channel and Semrock, FF01‐525/39‐25 for the green channel) before being focused onto a single‐photon avalanche diode (MPD, PD‐050‐CTC). The TCSPC and FCS data were collected using a time‐resolved module (PicoQuant, Multiharp 150 4P) with a time resolution of 5 ps. To validate system performance, focal volume calibration was conducted using Alexa Fluor 488 (NHS Ester powder, HPLC purity 98%, 1 mg, catalog number: A20000, Thermo Fisher Scientific) and Alexa Fluor 647 (NHS Ester powder, HPLC purity 98%, 1 mg, catalog number: A20006, Thermo Fisher Scientific) in aqueous solution, both with known diffusion coefficients. Burst thresholding was applied based on the photon count distributions to distinguish single‐vesicle fluorescence bursts from background noise.

### 4.9 FCCS Measurements

Each experiment began with system calibration using free Alexa 488 and Alexa 647 in solution to determine the photon count rates for the green and red channels. For dual‐color measurements, the EV membranes were stained with miRFP immediately beforehand. Then, 10 µL of the stained sample was placed on a glass slide over the microscope objective lens. All fluorescence recordings were conducted at a controlled temperature in a darkened room. To gather robust statistics across the EV size distribution, the measurements were repeated for each hiPSC condition.

### 4.10 FCCS Analysis

Because molecules freely diffuse in and out of the observation volume, the fluorescence intensity fluctuates. The autocorrelation function *G*(τ) for each red and green channel is computed from the fluorescence fluctuations δ*F* (*t*) =  *F*(*t*) − 〈*F*(*t*)〉, where 〈*F*(*t*)〉 is the mean fluorescence intensity.

(3) $$G\;\left(\tau \right)=\frac{\langle \delta F\left(t\right)\delta F\left(t+\tau \right)\rangle }{\langle F{\left(t\right)}^{2}\rangle }$$

Here, τ represents the lag time, and the angle brackets indicate a time‐based average. In FCCS, the cross‐correlation function *G_cross_* (τ) is obtained by analyzing the synchronized two‐color fluorescence intensity traces, thus quantifying the correlated fluctuations between signals from two spectrally distinct species.

(4) $$G_{cross}\left(\tau \right)=\frac{\langle \delta F_{r}\left(t\right)\delta F_{g}\left(t+\tau \right)\rangle }{\langle F_{r}\left(t\right)F_{g}\left(t\right)\rangle }\;$$

where *F_r_* (*t*) and *F_g_* (*t*) denote the time‐resolved fluorescence intensity traces recorded in the red and green channels, respectively.

### 4.11 Dual‐Color Coincident‐Burst Analysis

The burst analysis was performed through a search of the green and red bursts within the fluorescence time traces. The FRETBurst library (https://github.com/OpenSMFS/FRETBursts) in Python was used, which made it possible to determine the fluorescence burst parameters, including duration, intensity, and time occurrence, for both signal channels based on the analysis of photon time tags. The data of the photon time tags were time‐gated according to their excitation pulses to minimize the crosstalk between channels. To reveal the bursts, the values of *K_r_*, *K_g_*, *m_r_*, and *m_g_* were selected for the fluorescence signal data from undifferentiated hiPSC EVs to adjust the median values of the burst duration distribution close to the corresponding FCCS data. Here, *K_r_* and *K_g_* correspond to the burst intensity factors exceeding the background levels of the red and green signals, respectively, and *m_r_* and m_g_ denote the number of consecutive photons used to deduce the burst intensities of the red and green signals, respectively. The values of *K_r_·B_r_*, *K_g_·B_g_*, *m_r_*, and *m_g_* were fixed for all EV measurements, whereas *K_r_* and *K_g_* were modified according to the background values within each acquisition. Based on the time spans between photons arriving in bursts, the method was used to determine the burst intensity and total burst duration. A coincidence search was performed by checking whether a burst time occurrence in one detection channel was within the tolerance window of any burst time occurrence in the other channel. The burst rate was at all times less than 5.5 s^−1^ with a duration typically below 10 ms, which confirmed that the probability of detecting more than one burst (EV) was negligible. The tolerance window was selected based on the polydispersity of the EV size distribution. It was half the difference between green and red burst durations of 10% quantile from all detected bursts. This condition preserves nearly total overlap of the burst time ranges from the green and red channels. The total acquisition time of the EV fluorescence time traces was approximately 2.5 h for each hiPSC condition to capture tens of thousands of bursts in both detection channels. To perform statistical *t*‐tests, the time traces were split into subsections with 20 coincident bursts each, and the coincident‐burst rate and loading yield in those data subsets were deduced. The resulting data arrays were input into a statistical *t*‐test function. Statistical *t*‐tests of the null hypothesis verifying whether the coincident green burst rates and EV loading yields have the same mean values under different iPSC stress conditions were performed in SciPy library of Python. Welch's *t*‐test was applied to reject the null hypothesis that the undifferentiated hiPSC EVs and partially differentiated/amyloid‐β‐treated hiPSC EVs produce statistically identical coincident green burst rates and EV loading yields.

## Author Contributions

I.K., A.B., and D.D.N. conceptualized and designed the study. A.B. and D.D.N. developed the coincident‐fluorescence‐burst analysis platform and optimized the dual‐color fluorescence cross‐correlation spectroscopy setup. D.D.N. performed FCCS measurements. W.J.Y. and J.‐C.P. prepared and validated secreted extracellular vesicles from hiPSCs. All authors wrote and edited the manuscript and contributed to data analysis and discussion. I.K., J‐C.P. and A.B. guided the entire project.

## Funding

National Research Foundation (NRF) grants (RS‐2023‐00266110 and RS‐2024‐00462912) funded by the Ministry of Science and ICT (MSIT) of the Korean government. NRF Sejong Science Fellowship (RS‐2021‐NR061797, RS‐2022‐NR072469) funded by the MSIT of the Korean government. Ministry of Science and Higher Education of the Russian Federation (Agreement No. 075‐15‐2025‐017). Korea Dementia Research Project (RS‐2024‐00339665) through the Korea Dementia Research Center (KDRC), funded by the Ministry of Health & Welfare and MSIT. SKKU Academic Research Support Program (Samsung Research Fund), funded by Sungkyunkwan University.

## Conflicts of Interest

The authors declare no conflicts of interest.

## Supporting information




**Supporting File**: advs73468‐sup‐0001‐SuppMat.docx.

## Data Availability

The data that support the findings of this study are available from the corresponding author upon reasonable request.
