# Supplementary material for: Coincident Fluorescence‐Burst Analysis of Actin Cargo Molecules in Secreted Single Diffusing Extracellular Vesicles From Human Induced Pluripotent Stem Cells
Source: Adv Sci (Weinh). 2025 Dec 29;13(13):e14421. doi: 10.1002/advs.202514421 (PMC12955927; doi:10.1002/advs.202514421)
Supplement: Supplementary file 1 — Supporting File: advs73468‐sup‐0001‐SuppMat.docx. [file ADVS-13-e14421-s001.docx]

Supporting Information

Coincident Fluorescence-Burst Analysis of Actin Cargo Molecules in Secreted Single Diffusing Extracellular Vesicles from Human Induced Pluripotent Stem Cells

Dang Du Nguyen^#^, Aleksandr Barulin^#,^*, Won Jong Yu^#^, Jong-Chan Park* and Inki Kim*

**
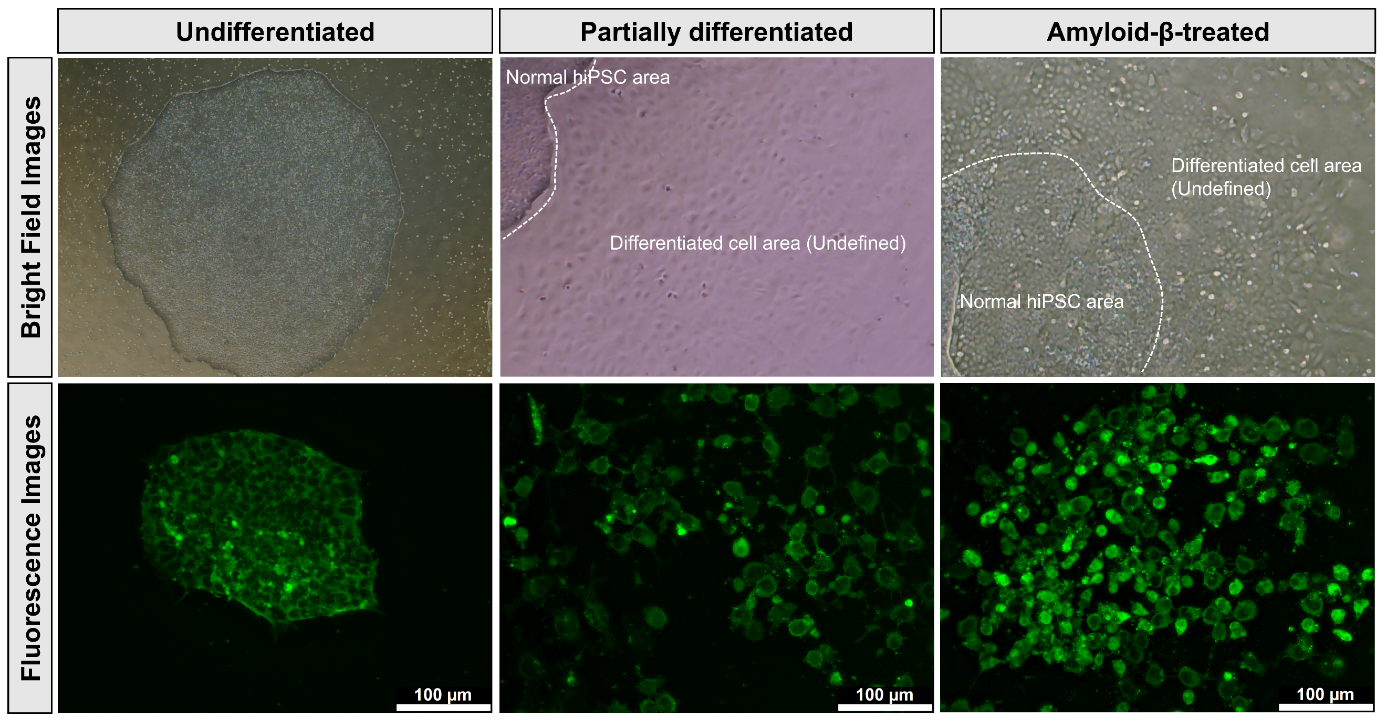
**

**Figure S1. Expression of actin-EGFP inside EVs with different conditions of hiPSCs.**

**
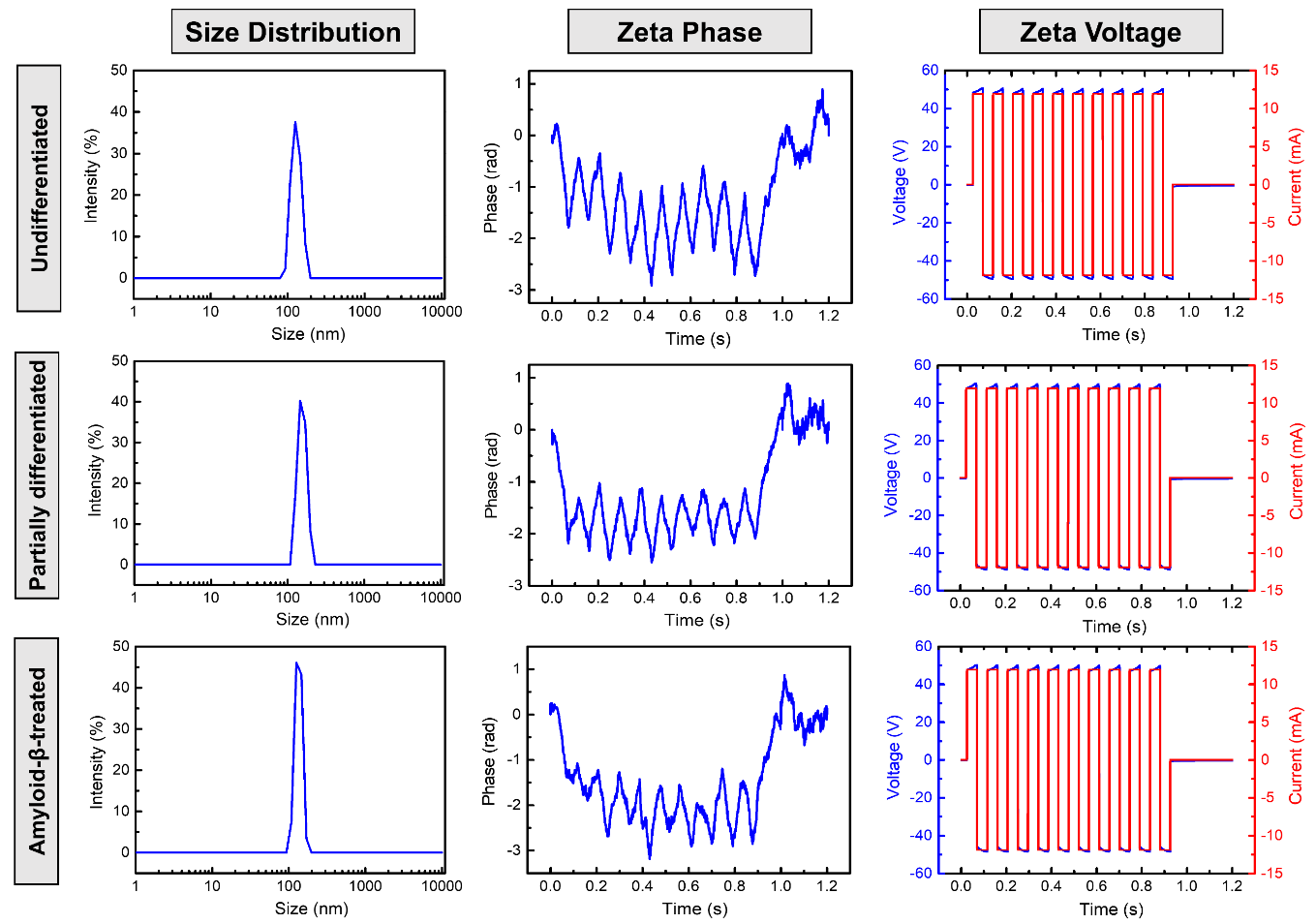
**

**Figure S2. EV characterization using DLS with different conditions of hiPSCs.**


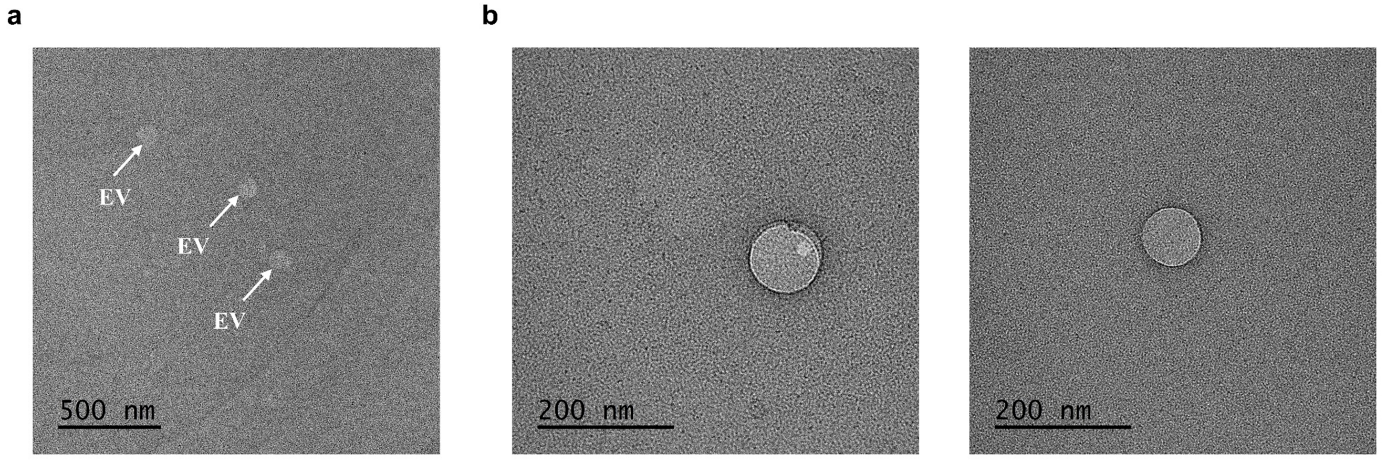


**Figure S3**. TEM images of isolated actin-EGFP-containing EVs with low magnification (a) and high magnification (b).

**
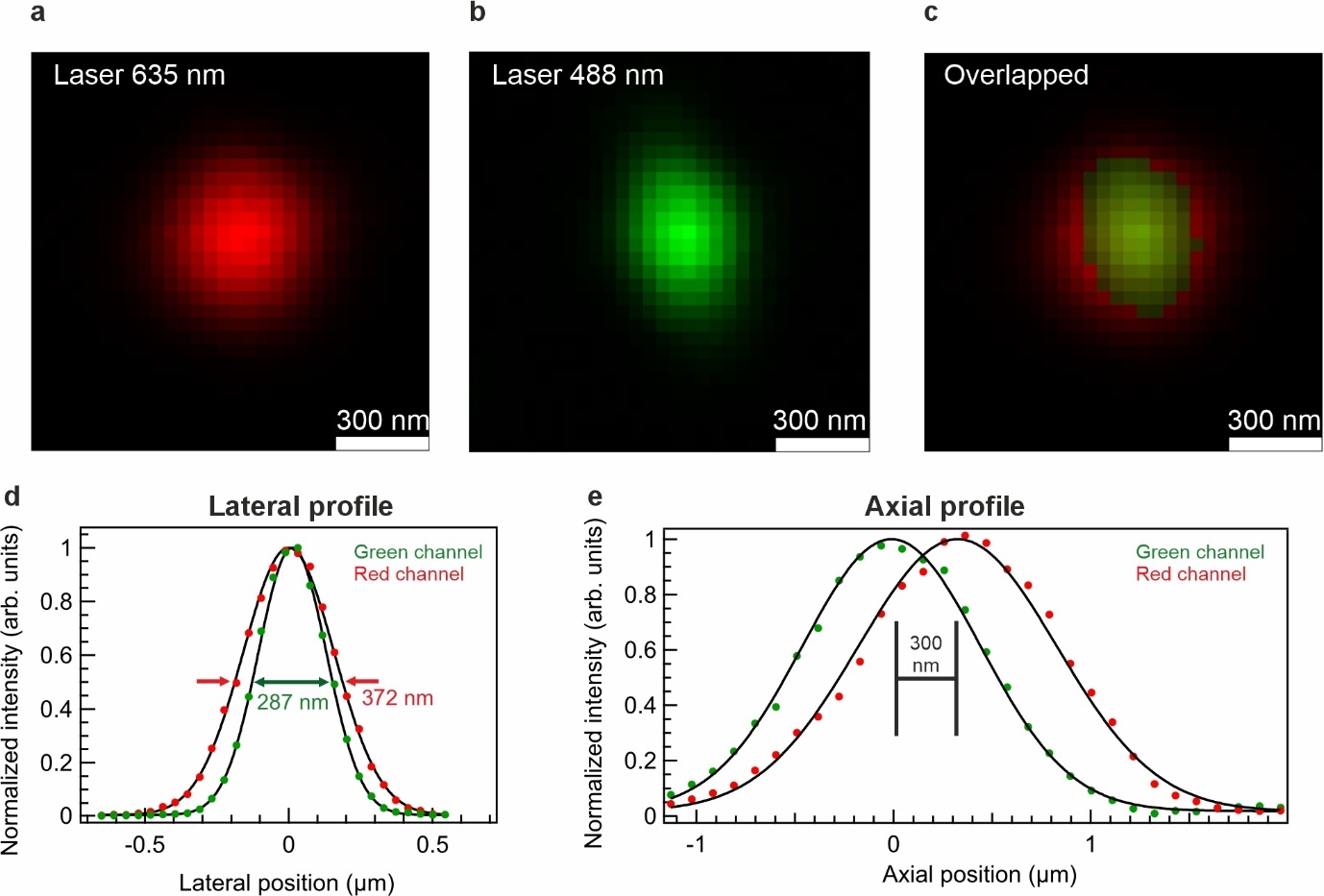
**

**Figure S4. Visualization of excitation beam overlap.** a) Fluorescence image of a multi-color-stained polysterene bead with diameter of 110 nm monitored in the red detection channel. b) Fluorescence image of a multi-color-stained polysterene bead with diameter of 110 nm monitored in the green detection channel. c) Overlapped images (a) and (b). d) Lateral intensity profiles for red and green detection volumes. e) Axial intensity profiles for red and green detection volumes.

**Supporting Text 1. Cross-correlation function analysis**

The number of dual-color emitters (*N_rg_*) is quantified via correcting background values as follows:^[1,2]^

| $G_{rg}^{meas}\left( \tau\right)=G_{rg}^{true}\left( \tau\right)\frac{F_{r}-B_{r}}{F_{r}}\cdot\frac{F_{g}-B_{g}}{F_{g}} ,$ | (S1) |
| --- | --- |
| $G_{rg}^{true}\left( 0 \right)=\frac{N_{rg}}{N_{r}\cdot N_{g}} ,$ | (S2) |

where $G_{rg}^{meas}\left( \tau\right)$ and $G_{rg}^{true}\left( \tau\right)$ are measured and true cross-correlation functions with *F_r_*, *B_r_* being fluorescence and background values of red fluorescence channel, and *F_r_*, *B_r_* being fluorescence and background values of green fluorescence channel. *N_rg_*, *N_r,_ N_g_* denote number of emitters with dual-color tags (*e.g.* loaded EVs), $N_{r}$ – number of red emitters, $N_{g}$ – number of green emitters. Through the determination of numbers of one-color emitters in each channel via autocorrelation functions in each channel and the true cross-correlation amplitude, the absolute values of the number of dual-color emitters are deduced.


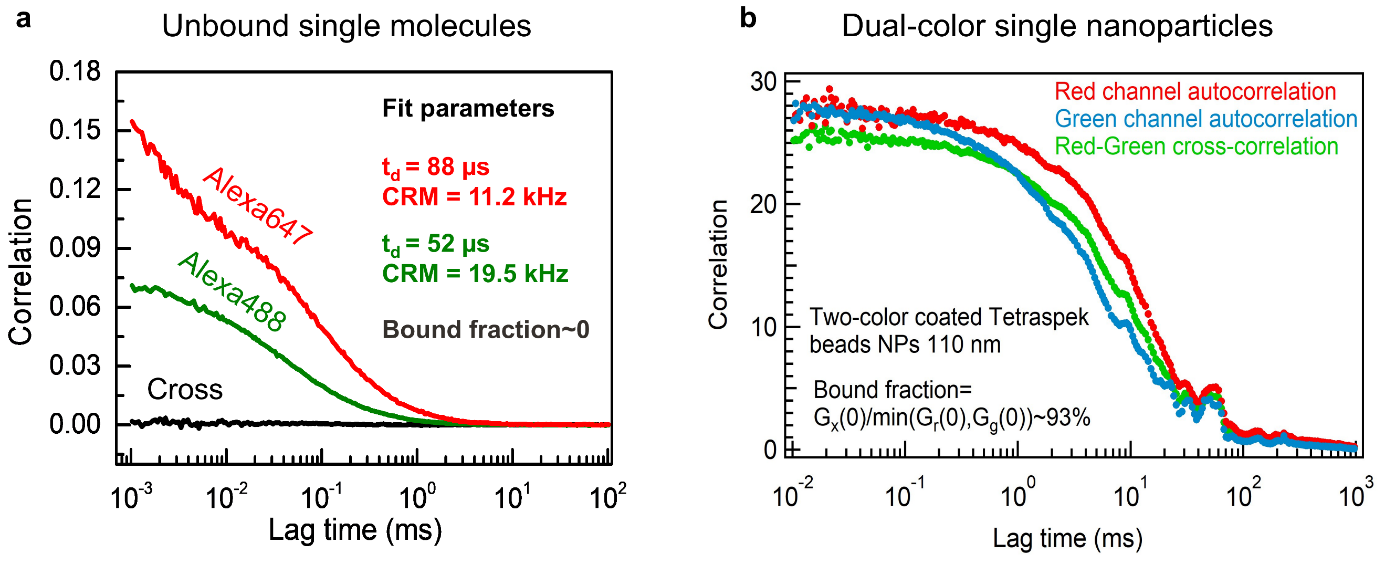


**Figure S5. Diffusion auto- and cross-correlation of unbound fluorophores and dual-color-stained nanoparticles.** a) Auto- and cross-correlation functions of diffusing Alexa Fluor 488 and Alexa Fluor 647 in mixture. b) Auto- and cross-correlation functions of multi-color-stained fluorescent nanoparticles.

**Table S1.** **FCCS fit parameters of diffusing actin-EGFP containing EVs derived from hiPSCs under different conditions.**

| **hiPSC conditions** | | **Correlation function curve** | ***G(0)*** | ***τ_D1_* (ms)** | ***τ_D2_* (ms)** | ***τ_D3_* (ms)** | ***F* (kcnts/s)** | ***B* (kcnts/s)** |
| --- | --- | --- | --- | --- | --- | --- | --- | --- |
| Undifferentiated | | Red | 43 | 0.08 | 2.4 |  | 0.37 | 0.16 |
|  | | Green | 8.1 | 0.02 | 0.07 |  | 0.16 | 0.09 |
|  | | Cross | 0.19 | 2.2 |  |  |  |  |
| Partially differentiated | | Red | 55.5 | 0.23 | 10 |  | 0.34 | 0.14 |
|  | | Green | 7.1 | 0.05 | 0.06 |  | 0.26 | 0.12 |
|  | | Cross | 0.29 | 11 |  |  |  |  |
| Amyloid-β treated | | Red | 33.44 | 3.9 | 4.9 | 6.1 | 0.35 | 0.27 |
|  |  | Green | 1.55 | 0.06 | 4 |  | 0.42 | 0.27 |
|  |  | Cross | 0.46 | 33 |  |  |  |  |

**Table S2.** **Burst search parameters within fluorescence time traces of diffusing actin-EGFP containing EVs.**

| **Detection channel** | **hiPSC conditions** | **K_r/g_‧B_r/g_ (kcnts/s)** | **K_r/g_** | **m_r/g_** |
| --- | --- | --- | --- | --- |
| Red | Undifferentiated | 1.303 | 16.1 | 10 |
|  | Partially differentiated | | 11.3 |  |
|  | Amyloid-β treated | | 9.7 |  |
|  |  |  |  |  |
| Green | Undifferentiated | 0.864 | 9.4 | 4 |
|  | Partially differentiated | | 5.4 |  |
|  | Amyloid-β treated | | 7.3 |  |


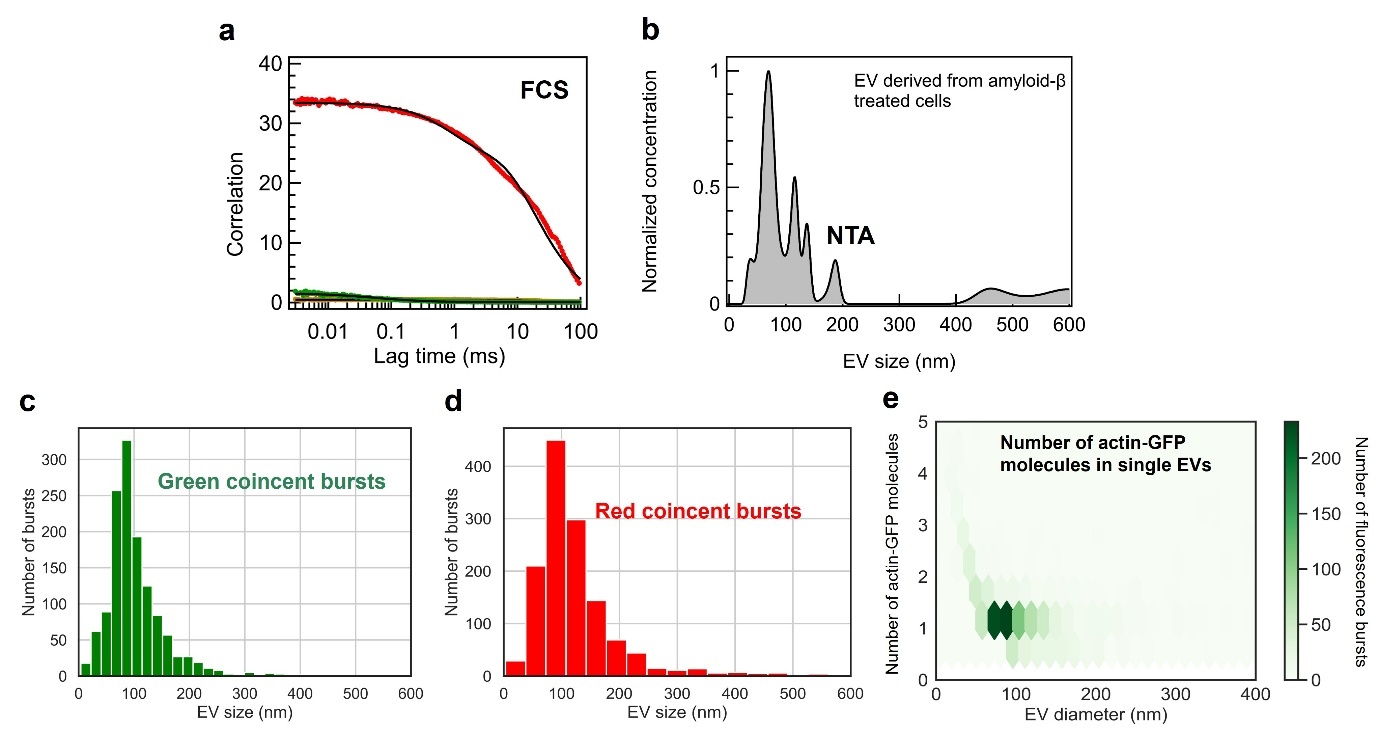


**Figure S6. Dual-color fluorescence analysis from single EVs derived from amyloid-β-treated hiPSCs.** a) Auto- and cross-correlation functions. b) Nanoparticle tracking analysis of size distribution. c) Size distribution based on green coincident burst analysis. d) Size distribution based on red coincident burst analysis. e) 2D histogram of number of actin-EGFP cargo molecules in single EVs derived from amyloid-β-treated hiPSCs.


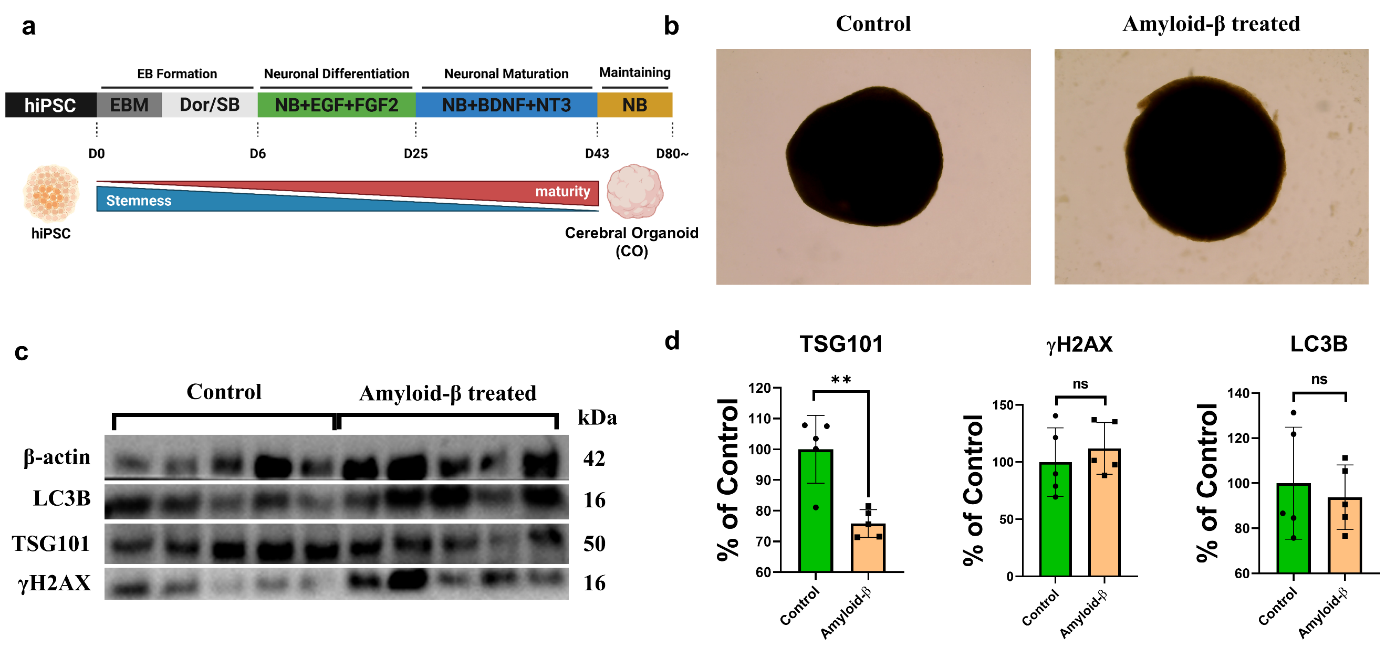


**Figure S7. The generation of cerebral organoids (CO) generation and assessment for the modeling of pathogenesis related condition.** a) Schematic of hiPSC-derived cerebral organoid (CO). b) Bright Field image of control CO and amyloid-β-treated CO. Each CO were matured until Day 110 and Amyloid-β-treatment were performed under the condition 2 μM of oligomerized amyloid-β for overnight. c) Western blot image of control CO and amyloid-β-treated CO. each condition was composed by *n* = 5. d) Comparison graph based on the western blot image. Each graph were analyzed by student’s T-test, and the correction test was performed by Mann-Whitney’s test. ns; non-significant, ***p<0.005.*


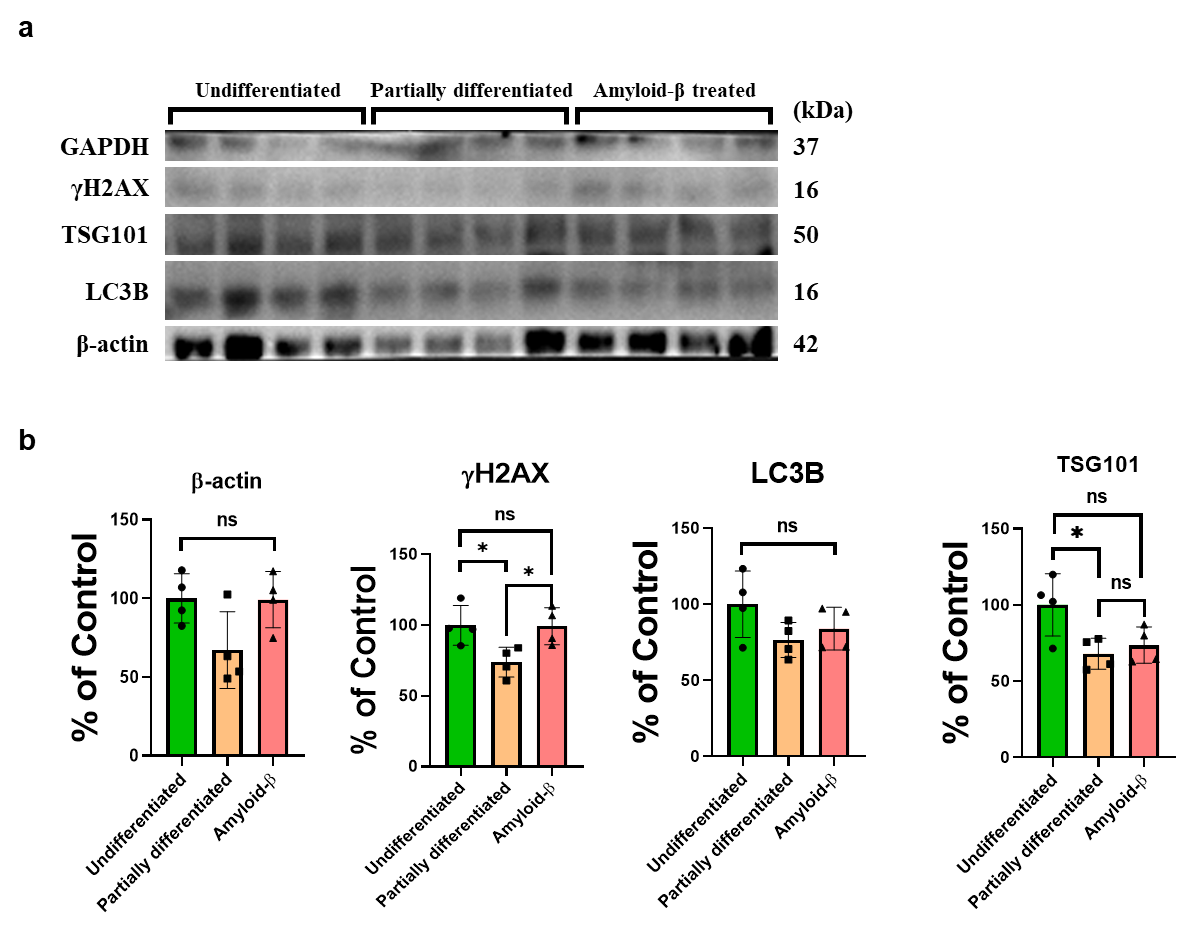


**Figure S8. The fluctuation of molecular biological characteristics of hiPSCs after exposed under stress-inducible condition.** a) The western blot image from each condition of hiPSCs group. b) Comparison graph acquired from western blot image of a) Each target band was analyzed statistically by One-Way ANOVA with the non-parametric test. Post-hoc correction were performed by Kruskal-Wallis test. ns; non-significant, *; *p<0.05,* **, *p<0.005*

**
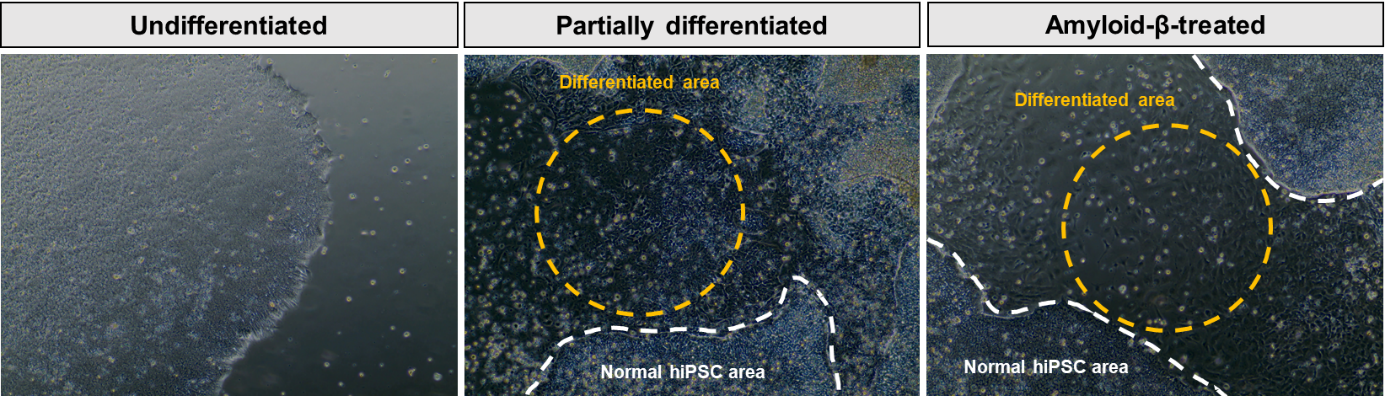
**

**Figure S9. Assessment of the effectiveness of EVs-derived from partially differentiated and amyloid-β-treated hiPSCs.** Undifferentiated healthy hiPSCs before the treatment of partially differentiated hiPSC-derived EVs (left). Partially differentiated hiPSCs after treatment of partially differentiated hiPSC-derived EVs (middle). Partially differentiated hiPSCs after treatment of amyloid-β-treated hiPSC-derived EVs (right). Each white line indicates the hiPSCs that is maintaining the stemness relatively compared with surrounded environment. Yellow circles indicate differentiated hiPSCs, which cell type is undefined.

**References**

[1] L. Yu, Y. Lei, Y. Ma, M. Liu, J. Zheng, D. Dan, P. Gao, *Front. Phys.* **2021**, *9*, 644450.

[2] K. Bacia, S. A. Kim, P. Schwille, *Nat. Methods* **2006**, *3*, 83.
